# Identification of nafamostat mesylate as a selective stimulator of NK cell IFN-γ production via metabolism-related compound library screening

**DOI:** 10.1007/s12026-022-09266-z

**Published:** 2022-02-15

**Authors:** Qinglan Yang, Shuju Zhang, Shuting Wu, Baige Yao, Lili Wang, Yana Li, Hongyan Peng, Minghui Huang, Qinghua Bi, Peiwen Xiong, Liping Li, Yafei Deng, Youcai Deng

**Affiliations:** 1 Pediatrics Research Institute of Hunan Province, Hunan Children’s Hospital, Changsha, 410007 China; 2 Pediatric Intensive Care Unit, Hunan Children’s Hospital, University of South China, Changsha, 410007 China; 3 Department of Pharmacy, The Third Xiangya Hospital, Central South University, Changsha, 410013 China; 4 Institute of Materia Medica, College of Pharmacy, Army Medical University (Third Military Medical University), Chongqing, 400038 China

**Keywords:** Natural killer cell, IFN-γ, Statins, Nafamostat mesylate

## Abstract

Natural killer (NK) cells play important roles in controlling virus-infected and malignant cells. The identification of new molecules that can activate NK cells may effectively improve the antiviral and antitumour activities of these cells. In this study, by using a commercially available metabolism-related compound library, we initially screened the capacity of compounds to activate NK cells by determining the ratio of interferon-gamma (IFN-γ)^+^ NK cells by flow cytometry after the incubation of peripheral blood mononuclear cells (PBMCs) with IL-12 or IL-15 for 18 h. Our data showed that eight compounds (nafamostat mesylate (NM), loganin, fluvastatin sodium, atorvastatin calcium, lovastatin, simvastatin, rosuvastatin calcium, and pitavastatin calcium) and three compounds (NM, elesclomol, and simvastatin) increased the proportions of NK cells and CD3^+^ T cells that expressed IFN-γ among PBMCs cultured with IL-12 and IL-15, respectively. When incubated with enriched NK cells (purity ≥ 80.0%), only NM enhanced NK cell IFN-γ production in the presence of IL-12 or IL-15. When incubated with purified NK cells (purity ≥ 99.0%), NM promoted NK cell IFN-γ secretion in the presence or absence of IL-18. However, NM showed no effect on NK cell cytotoxicity. Collectively, our study identifies NM as a selective stimulator of IFN-γ production by NK cells, providing a new strategy for the prevention and treatment of infection or cancer in select populations.

**Supplementary Information:**

The online version contains supplementary material available at 10.1007/s12026-022-09266-z.

## Introduction

NK cells are a group I innate lymphoid cells (ILCs) that are capable of combating virus-infected and tumour cells [1]. The activation of NK cell effector function does not depend on RAG-dependent receptor rearrangement or antigen presentation but on the balance of the expression levels of inhibitory receptors and active receptors on the NK cell surface [2]. Activated NK cells can destroy tumour cells and virus-infected cells by direct cytolysis via degranulation. They can also secrete several cytokines, such as interferon gamma (IFN-γ) and tumour necrosis factor alpha (TNF-α), which participate in regulating the adaptive immune response [3]. NK cell-based immunotherapy has been widely used in cancer treatments, including adoptive transfer of autologous and allogenic NK cells, checkpoint receptor blockade, and applications involving memory-like NK cells and chimeric antigen receptor (CAR) NK cells [4, 5]. Therefore, the identification of new approaches that can activate the effector function of NK cells is of great significance for immunotherapy of cancer and virus infection.

IFN-γ is a type 2 cytokine that plays important role in tissue homeostasis, immune and inflammatory responses, and tumour and virus immunosurveillance [6, 7]. NK cells are the primary robust source of IFN-γ prior to the arrival of adaptive immune cells that play critical roles in controlling virus [1] and bacterial infections [8]. Previous studies have demonstrated that IFN-γ production by NK cells in the peripheral blood can act as a supportive diagnostic or prognostic marker for some cancers, such as gastric and non-small cell lung cancer [9, 10]. Therefore, the identification of new molecules that can activate NK cell IFN-γ production is useful for improving NK cell-based immunotherapy.

Recently, studies have shown the importance of metabolism in promoting NK cell effector functions, including cytolytic functions and IFN-γ production [11–13]. A range of molecules, such as Rfx7 [14] and Srebp [15], control NK cell metabolism, thereby affecting NK cell cytotoxicity and IFN-γ production. Thus, in this study, we selected a commercially available metabolism-related compound library that includes compounds related to glucose metabolism, lipid metabolism, proteolysis, nucleotide metabolism, and other metabolic pathways in the hope that some of the metabolism-related compounds would be able to activate NK cells.

Upon encountering infected or transformed cells, antigen-processing cells can produce large amounts of IL-12 and IL-15, which are critical for activating both innate and adaptive immune responses [16]. IL-12 and IL-15 are two representative type I cytokines that are critical for NK cell activation and effector functions and can induce IFN-γ production by NK cells [17]. As NK cells produce very low levels of IFN-γ without cytokine stimulation, the presence of low concentrations of IL-12 or IL-15 is necessary to better and accurately evaluate the percentage of IFN-γ^+^ NK cells in human peripheral blood mononuclear cells (PBMCs) by flow cytometry. To this end, we initially determined the capacity of metabolism-related compounds to regulate IFN-γ production by NK cells in the presence of IL-12 or IL-15. We identified that a synthetic serine protease inhibitor, nafamostat mesylate (NM), could selectively enhance NK cell IFN-γ production but not cytolytic function.

## Materials and methods

### Metabolism-related compound library

The metabolism-related compound library (L3700-Z303367), a unique collection of 513 candidate compounds, was purchased from Selleckchem (Houston, TX, USA). Additional NM was purchased from Selleckchem (S1386).

### Isolation of PBMCs and NK cells

PBMCs and NK cells were isolated from the peripheral blood from healthy donors. Isolation of PBMCs through Ficoll-Paque PLUS (GE Healthcare Biosciences, Pittsburgh, PA, USA) density gradient centrifugation was performed as described previously [18]. Enriched NK cells were obtained from PBMCs using a MACSxpress NK cell isolation kit (Miltenyi Biotec, San Diego, CA, USA), and the resulting purity was ≥ 80.0%. The enriched NK cells were further purified by fluorescence-activated cell sorting (FACS) using a FACSAria III cell sorter (BD Biosciences, San Jose, CA, USA) after gating on CD56^+^CD3^−^ cells, as described previously [18]. The purity of the purified CD56^+^CD3^−^ NK cells was ≥ 99.0%.

### Cell culture and treatments

PBMCs, NK cells, and K562 cells were cultured or maintained in RPMI-1640 medium (Invitrogen, Carlsbad, CA, USA) supplemented with 10% heat-inactivated foetal bovine serum (FBS), penicillin (100 U/mL), and streptomycin (100 mg/mL) (Sigma, St. Louis, MO, USA) at 37 °C in 5% CO_2_.

For the stimulation of PBMCs (1 × 10^6^/well), enriched NK cells (2 × 10^5^/well) or purified NK cells (2 × 10^5^/well), cells in 200 μL were seeded into a 96-well culture plate and treated with DMSO or the indicated chemicals (10 μM) for 24 h in the presence of human recombinant IL-12 (10 ng/mL), IL-15 (10 ng/mL), or IL-18 (10 ng/mL). Then, the cells were harvested flow cytometry analysis, and the supernatants were harvested for enzyme-linked immunosorbent assay (ELISA).

### Flow cytometric analysis

Flow cytometry was performed as previously described [19]. For the intracellular staining of IFN-γ, GolgiPlug, and GolgiStop (BD Biosciences) were added to cells 4 h before harvest. Then, the cells were surface stained with anti-CD3-PECy7 and anti-CD56-APC antibodies (BD Biosciences), washed, and resuspended in Cytofix/Cytoperm solution (BD Biosciences), and incubated at 4 °C for 25 min. Finally, the cells were stained with anti-IFN-γ-FITC antibodies (BD Biosciences). For surface staining, including staining for NCR3 (also called NKp30), killer cell lectin-like receptor K1 (KLRK1, also called NKG2D), Fc fragment of IgG receptor IIIa (also called CD16), TNF-related apoptosis-inducing ligand (TRAIL), killer cell lectin-like receptor C1 (KLRC1, also called NKG2A), factor-associated suicide ligand (FasL), and CD14, the cells were stained with the above antibodies (Biolegend, San Diego, CA, USA) and incubated at room temperature in the dark for 15 min. The samples were analysed with an LSRFortessa flow cytometer (BD Biosciences), and FlowJo software (Tree Star, Ashland, OR, USA) was used to analyse the data.

#### ELISA

The secretion of interferon-γ into the supernatant of these cells was assessed by an ELISA kit (Biolegend) according to the manufacturer’s protocol. The absorbance was read at 450 and 570 nm using dual wavelengths.

### NK cell cytotoxicity assay

The CD107a expression assay was performed as previously described [20]. Purified NK cells (5 × 10^5^/well) were preactivated with different concentrations of NM (25 μM) alone or in the presence of IL-12, IL-15, or IL-18 for 18 h. Then, the preactivated NK cells were mixed with K562 cells (1 × 10^5^/well) at a 5:1 E:T ratio in a V-bottom 96-well plate in the presence of anti-CD107a antibody (BD Biosciences), GolgiPlug and GolgiStop, and the cells were cocultured for another 5 h. Then, the cells were collected to measure CD107a expression by flow cytometry (LSRFortessa, BD Biosciences).

For the K562 cell apoptosis assay, purified human primary NK cells (5 × 10^5^/well) were preactivated with 25 μM NM or DMSO for 18 h in 96-well V-plates. The K562 cells were stained with 5 μM CTV dye in RPMI 1640 medium (Thermo Fisher Scientific, Waltham, MA, USA) for 20 min at 37 °C, added to wells containing activated NK cells at a 5:1 ratio, and cocultured for another 5 h. The cells were then harvested and stained with annexin V and 7-AAD (apoptosis kit, BD Biosciences) and analysed by flow cytometry [21]. The ratio of annexin V^−^7-AAD^−^ cells among the CTV^+^ cells was determined and represented the ratio of the remaining live target cells.

### Statistical analysis

A paired *t*-test was used to compare two conditions with repeated measures from the same donor. The Friedman test was used for multiple related comparisons. All the statistical analyses were performed using SPSS version 18 (IBM, NY, USA). All *p*-values were 2-sided, and *p* < 0.05 was considered significant for all tests.

## Results

### Screening of compounds that induce primary human NK cell IFN-γ production

**Fig. 1:**
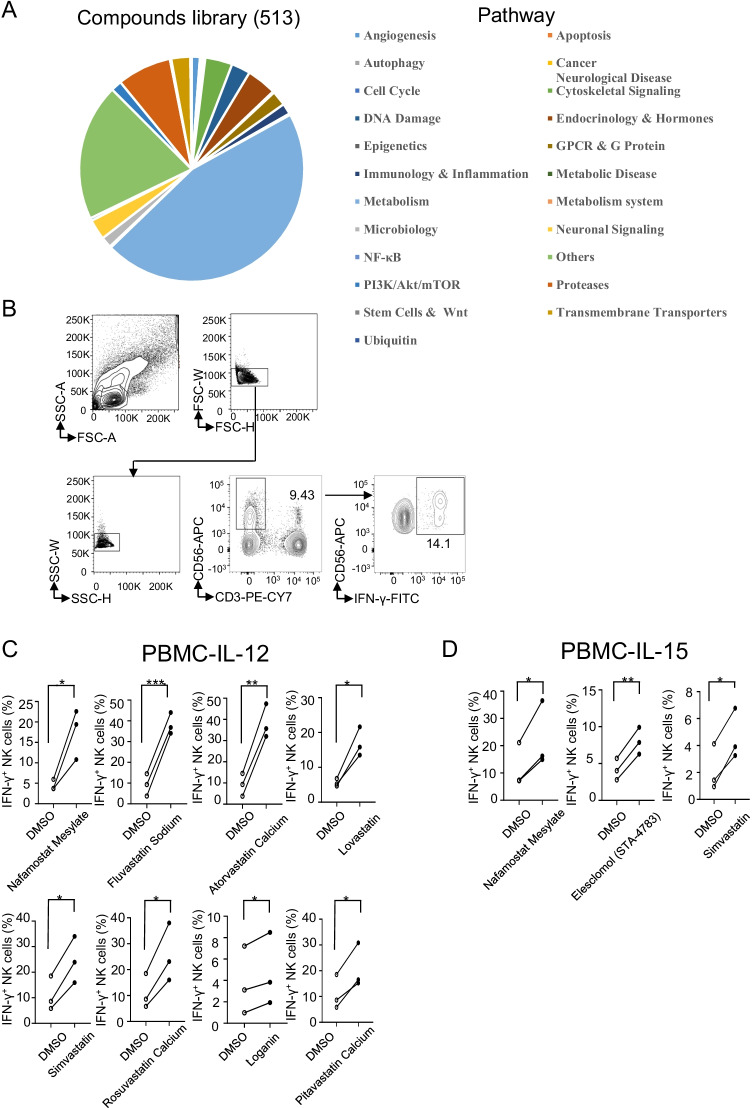
Screening of compounds capable of activating primary human NK cells by assessing the proportion of IFN-γ^+^ cells in the NK cell population of PBMCs. **A** Summary of 513 compounds based on the molecular pathways they affect. **B** Gating strategy used to identify IFN-γ-producing NK cells in healthy donor PBMCs treated with DMSO or the compounds. **C, D** Flow cytometry analysis of IFN-γ^+^ NK cells among healthy donor PBMCs treated for 18 h with DMSO or the indicated compounds in the presence of IL-12 (10 ng/mL) (**C**) or IL-15 (10 ng/mL) (**D**). Each dot represents one donor (three donors are used for each compound), and dots from two groups connected by a line represent two samples from the same donor. A paired *t*-test was used for **C** and **D**. *p < 0.05, **p < 0.01 compared with the DMSO group (**C, D**)

The metabolism-related compound screening library was purchased from Selleckchem and contained 513 candidate compounds. The compounds are involved in pathways including pathways related to angiogenesis, apoptosis, autophagy, cancer, neurological disease, cell cycle progression, cytoskeletal signalling, DNA damage, endocrinology and hormones, epigenetics, GPCRs and G proteins, immunology and inflammation, metabolic disease, metabolism, the metabolism system, microbiology, neuronal signalling, NF-κB, PI3K/Akt/mTOR, proteases, stem cells and Wnt, transmembrane transporters, and so on (Fig. 1A). We initially looked for an increase in the proportion of IFN-γ-producing NK cells in the presence of IL-12 or IL-15 by flow cytometry to screen for relevant compounds. The gating strategy used to identify IFN-γ-producing NK cells after healthy donor PBMCs were treated with DMSO or a compound is shown in Fig. 1B.

After an initial test with a sample from one donor, 71 candidates of the 513 compounds induced a greater than 50% increase in the proportion of IFN-γ^+^ cells in the NK cell population (CD3^−^CD56^+^) compared with IL-12 alone (Supplementary Fig. 1A). We next validated these 71 compounds by using PBMCs from three more donors, and the data revealed that eight candidates, including NM, fluvastatin sodium, atorvastatin calcium, lovastatin, simvastatin, rosuvastatin calcium, loganin, and pitavastatin calcium, consistently increased the proportion of IFN-γ^+^ cells in the NK cell population from almost all the tested donors (Fig. 1C). Similarly, the initial screening showed that 62 compounds caused a more than 50% increase in the proportion of IFN-γ^+^ cells in the NK cell population after the cells were cultured in the presence of IL-15 (10 ng/mL) for 18 h (Supplementary Fig. 1B). Furthermore, examination of samples from three more donors revealed that NM, simvastatin, and elesclomol (STA-4783) increased the proportion of IFN-γ^+^ NK cells (Fig. 1D).

In addition, we also explored whether NM affects IFN-γ production by T cells in PBMCs. Flow cytometry analysis revealed that NM, fluvastatin sodium, atorvastatin calcium, lovastatin, and loganin increased the proportion of IFN-γ^+^ cells in the CD3^+^CD56^−^ T cell population in the presence of IL-12 (Supplementary Fig. 2A), whereas NM and simvastatin increased the proportion of IFN-γ^+^ cells in the CD3^+^ T cell population in the presence of IL-15 (Supplementary Fig. 2B).

Overall, we found that NM and simvastatin could increase the proportion of IFN-γ^+^ cells in both the NK cell and T cell populations in PBMCs in the presence of IL-12 or IL-15.

### Effects of the nine identified candidates on IFN-γ levels secreted into the supernatants of cultured enriched NK cells

**Fig. 2:**
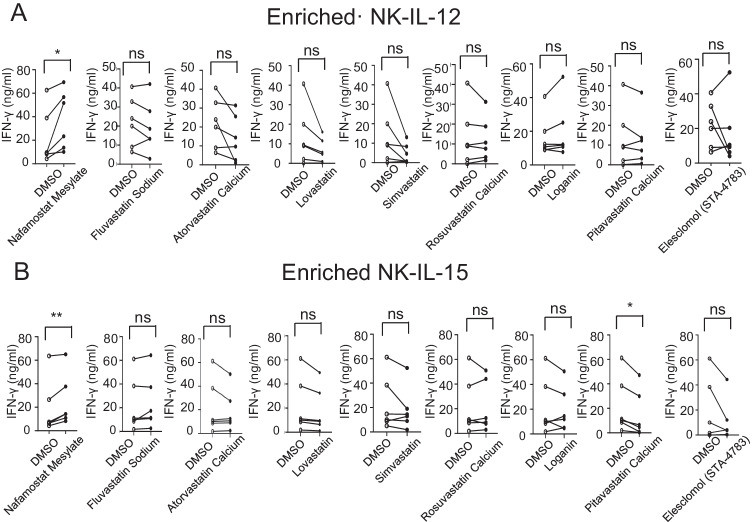
Effects of the nine identified candidates on levels of IFN-γ secreted into the supernatants of cultured enriched NK cells. **A**, **B** Enriched NK cells were treated with DMSO (control) or one of the nine compounds for 24 h in the presence of IL-12 (10 ng/mL) (**A**) or IL-15 (10 ng/mL) (**B**), and the protein levels of IFN-γ in the cell culture supernatants were detected with an ELISA kit (*n* = 6). The data were analysed by paired *t*-test. *, *p* < 0.05; **, *p* < 0.01; ns (not significant), *p* > 0.05 compared with the DMSO control group. Each dot represents one donor, and dots from two groups connected by a line represent two samples from the same donor

To further determine whether the nine tested candidates can promote IFN-γ secretion by NK cells, they were incubated with NK cells enriched from PBMCs in the presence of IL-12 or IL-15 for 24 h. We assessed IFN-γ secretion into the supernatants of the cultured enriched NK cells by ELISA. We found that NM enhanced IFN-γ secretion in the presence of IL-12, whereas the other eight compounds did not (Fig. 2A). In addition, in the presence of IL-15, only NM induced IFN-γ secretion, whereas pitavastatin calcium decreased IFN-γ secretion by NK cells. In addition, the other seven compounds caused no significant changes in the secretion of IFN-γ irrespective of the presence of IL-12 or IL-15 (Fig. 2B).

Flow cytometry analysis further validated that NM increased the percentage of IFN-γ^+^ NK cells in the enriched NK cell population after culture in the presence of IL-12 or IL-15 for 18 h (Figure S3A). We also measured IFN-γ secretion by ELISA after short-term NM stimulation for 6 h in the presence of IL-12 or IL-15. Our data revealed that NM treatment resulted in a trend of enhanced IFN-γ secretion by NK cells compared with DMSO, but the results were not statistically significant (Figure S3B). Among PBMCs, both CD56^bright^ and CD56^dim^ NK cells produced more IFN-γ after NM stimulation than after cytokine stimulation alone (Figure S3C). The enriched NK cell population also contains some contaminated T cells and monocytes; however, they produced almost no IFN-γ in response to the concentrations of cytokines we used (Figure S4).

These data suggest that NM may induce prolonged IFN-γ production in both CD56^bright^ and CD56^dim^ NK cells in the presence of IL-12 or IL-15.

### Effects of nafamostat mesylate on the expression of IFN-γ in purified NK cells

**Fig. 3:**
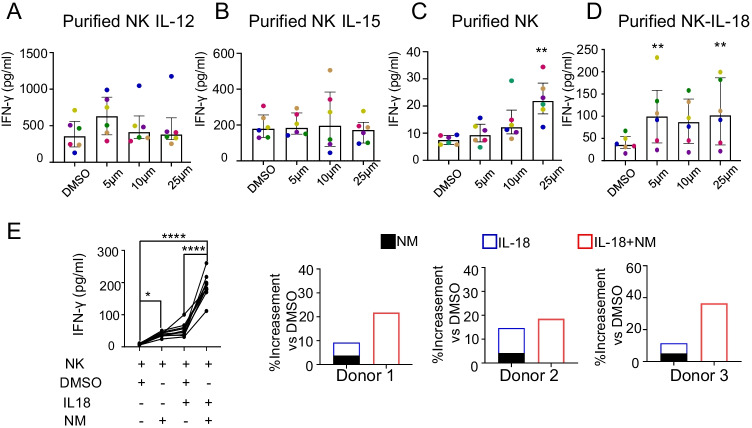
Effects of nafamostat mesylate on the expression of IFN-γ in purified NK cells. **A–D** Purified NK cells were treated with DMSO as a vehicle or the indicated concentration of NM in the presence or absence of different cytokines for 24 h. The protein levels of IFN-γ in the cell culture supernatants were detected with an ELISA kit (*n* = 6). **A** IL-12, 10 ng/mL; **B** IL-15, 10 ng/mL; **C** no cytokines; **D** IL-18, 10 ng/mL. The data were analysed by the Friedman test and are shown as the median ± interquartile range. *, *p* < 0.05; **, *p* < 0.01 compared with the DMSO control group (**A–D**). **E** Synergistic effect of NM and IL-18 on IFN-γ production. The same donor-derived NK cells (*n* = 10) were stimulated with DMSO, 25 μm NM, 10 ng/mL IL-18, or both IL-18 and NM for 24 h, and then the supernatants were analysed by ELISA. In each donor, the paired bars compare the additive effect of IL-18 and NM treatment alone (left, composite bar) versus the effect of costimulation with IL-18 and NM (right, black bar). Additive effect of IL-18 and NM versus costimulation with IL-18 and NM. *p* < 0.05

To further determine whether NM promotes IFN-γ secretion directly or indirectly, human NK cells purified from PBMCs were incubated with different concentrations (5, 10, or 25 μM) of NM in the presence of IL-12 or IL-15, followed by the measurement of IFN-γ secretion into the supernatants of cultured purified NK cells by ELISA. Unfortunately, NK cells only showed trends of increased IFN-γ secretion after NM treatment in the presence of IL-12 or IL-15, but the difference was not statistically significant (Fig. 3A, B).

We further explored whether NM alone could induce IFN-γ secretion by NK cells directly. The data showed that 5 to 25 μM NM alone increased IFN-γ secretion by NK cells in a concentration-dependent manner, and the results were statistically significant at 25 μM (Fig. 3C). Several studies have revealed that IL-18 exerts a synergistic effect with many cytokines, such as IL-12, IL-15, and INF-α, on IFN-γ production by NK cells [22, 23]. As NM alone could directly enhance IFN-γ production by purified human primary NK cells, we further investigated whether there was a synergistic effect between NM and IL-18 on inducing IFN-γ production by NK cells. In the presence of IL-18, NM increased IFN-γ secretion by NK cells, and the results were statistically significant at 5 μM and 25 μM (Fig. 3D). In an analysis of the data from the same donor-derived NK cells, 25 μM NM and 10 ng/mL IL-18 showed a synergistic effect on IFN-γ production (Fig. 3E).

Taken together, these results demonstrate that NM promotes NK cell IFN-γ production alone or in the presence of IL-18.

### Effects of nafamostat mesylate on cytotoxicity and the expression of germline-encoded receptors

**Fig. 4:**
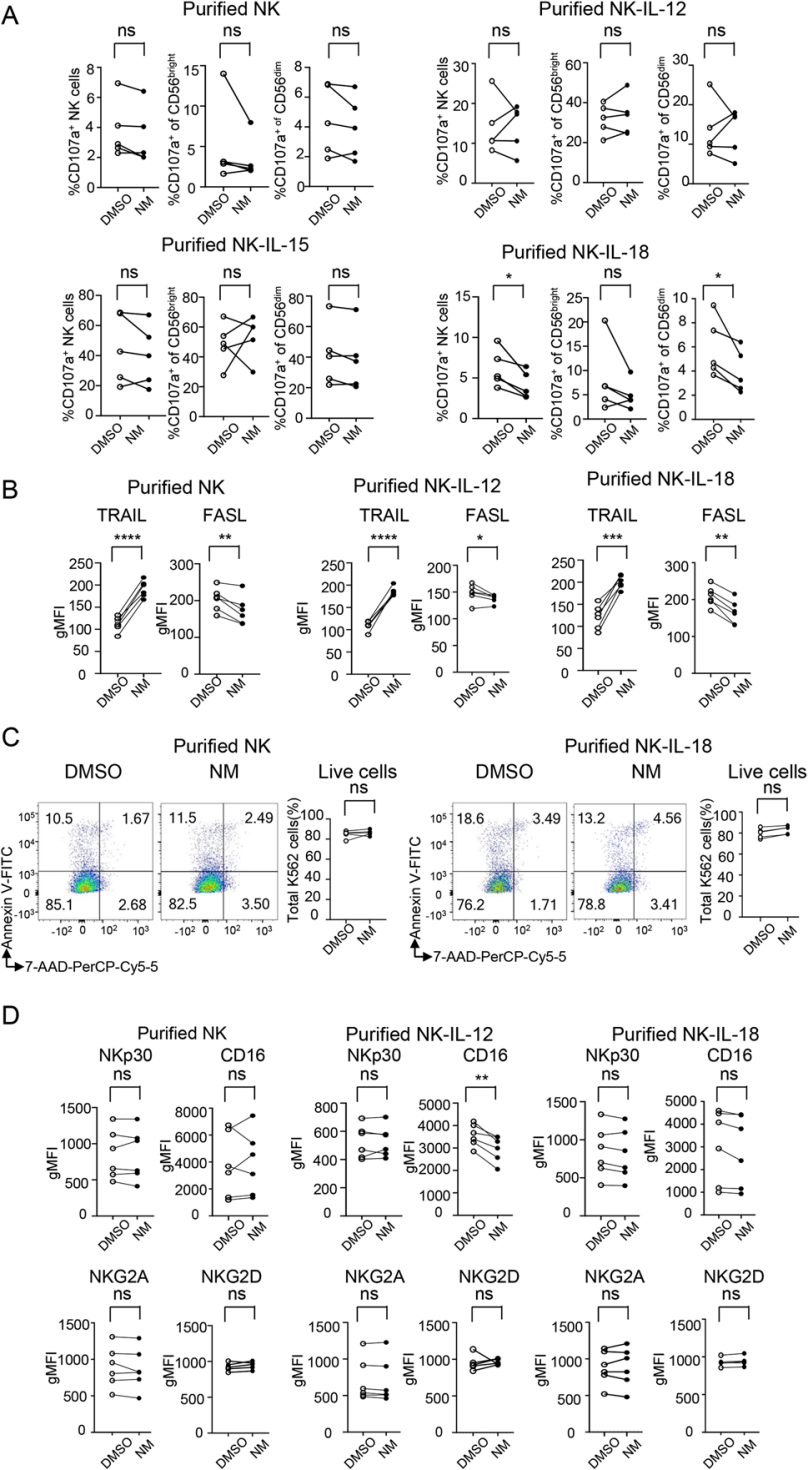
Effects of nafamostat mesylate on cytotoxicity and expression of germline-encoded receptors. **A** Purified NK cells were treated with DMSO or 25 μm NM alone or in the presence of IL-12 (10 ng/mL), IL-15 (10 ng/mL), or IL-18 (10 ng/mL) for 18 h and then mixed and incubated with K562 cells for another 5 h. The expression of CD107a was measured by flow cytometry. CD107a expression on both CD56^dim^ and CD56^bright^ NK cells is shown in the right two panels. Cumulative frequencies of CD107 are shown, and the corresponding representative flow plots are shown in Figure S5. **B, D** Purified NK cells were treated with DMSO or 25 μm NM alone or in the presence of IL-12 (10 ng/mL) or IL-18 (10 ng/mL) for 24 h, and then, the expression of TRAIL, FASL (**B**) and NKp30, NKG2D, CD16, and NKG2A (**D**) on NK cells was determined by flow cytometry. The gMFI for each marker is shown. The corresponding representative flow plots and cumulative frequencies are shown in Figure S5. **C** Purified NK cells were treated as described in **A**, and the ratio of live CTV-labelled K562 cells (annexin V^−^7-AAD^−^) was measured by flow cytometry. The data were analysed by paired t test. *N* = 5; *, *p* < 0.05; **, *p* < 0.01 compared with the DMSO control group (**A–D**)

Upon encountering target cells, NK cells exert their cytotoxic effects mainly through direct degranulation of perforin and granzyme B or activation of death receptor-related apoptosis via the production of TNF-related apoptosis-inducing ligand (TRAIL) and Fas ligand (FasL) [24]. CD107a (also called LAMP1) is a degranulation marker that is related to NK cell ability to lyse target cells [25]. Our data showed that NM had no obvious effect on the expression levels of CD107a in NK cells regardless of whether NM was administered in combination with IL-12, IL-15, or alone. Even at a higher concentration (25 μM), NM slightly reduced the expression of CD107a in NK cells, especially in CD56^dim^ NK cells, in the presence of IL-18 (Figs. 4A and S5A). The flow cytometry data showed that 25 µM NM significantly enhanced the expression levels of TRAIL but moderately reduced the expression of FASL in the presence or absence of IL-12 or IL-18 (Figs. 4B and S5B).

The above findings prompted us to find more direct evidence that NM regulates NK cell cytotoxicity by assessing the apoptosis of K562 cells after 5 h of coculture with purified human primary NK cells. The data revealed that the remaining percentage of live K562 (annexin V^−^7-AAD^−^) cells in the co-culture system were comparable between the systems that included NM- or DMSO-treated purified human primary NK cells, regardless of the presence or absence of IL-18 (Fig. 4C). These data suggest that NM does not affect final NK cell cytotoxicity because it reduces the protein levels of CD107a and FASL but increases the protein levels of TRAIL on NK cells.

Upon encountering target cells, NK cell effector functions are regulated by the balance of the expression levels of germline-encoded inhibitory receptors and activation receptors [26]. To find more evidence that NM affects NK cell cytotoxicity, we next measured the expression of activation receptors, including NKp30, NKG2D, and CD16, as well as inhibitory receptors, including NKG2A [26]. Consistent with the above finding that NM did not affect the overall cytotoxicity of NK cells, NM exerted no obvious effect on the protein levels of NKp30, NKG2D, CD16, and NKG2A (Figs. 4D and S5C).

Taken together, these results demonstrate that NM selectively promotes NK cell IFN-γ production without affecting cytolytic function.

## Discussion

NK cells are a critical subset of innate lymphocytes that combat tumour or virus-infected cells. Improving or rescuing NK cell effector function is a promising strategy for the prevention or treatment of cancer and viral infection. However, effective approaches for improving or rescuing NK cell effector function have yet to be realized. For example, although the administration of cytokines, such as IL-2, enhances NK cell effector function, IL-2 induces regulatory T cell expansion, which in turn dampens NK cell effector function [27, 28]. Therefore, the identification of molecules that can induce NK cell effector function with relative specificity will be useful for cancer or infection prevention.

Statins, a class of drugs that can inhibit 3-hydroxy-3-methylglutaryl coenzyme A (HMG-COA) reductase, have been approved for the treatment of hypercholesterolemia and prevent cardiovascular diseases [29]. In this study, we found that several statins could increase IFN-γ production by NK cells when they were incubated with PBMCs in the presence of IL-12. However, this effect was not observed in enriched NK cells activated with IL-12 or IL-15, which indicates an indirect role of these statins via the activation of other immune cell subsets. Consistently, statins have also been previously reported to indirectly enhance IFN-γ secretion by T lymphocytes, which is dependent on the IL-18 produced by other immune cells [30]. IL-12 and IL-18 are also potent stimulators of NK cell IFN-γ production [31]; therefore, we hypothesized that statin-induced increases in monocyte-derived cytokine production might be responsible for enhanced NK cell IFN-γ production in the PBMC population.

NM, a synthetic serine protease inhibitor [32], has been used to treat pancreatitis and acute vasculitis clinically in Japan. NM has been recently shown to be beneficial for patients with coronavirus disease 2019 (COVID-19) in some case reports [33–35]. In our study, we found that NM promoted NK cell IFN-γ production in both direct and indirect manners. Both the early burst of IFN-γ production by innate immune cells and the later sustained IFN-γ production by adaptive immune cells, such as T helper (TH) 1 cell and CD8^+^ cytotoxic T lymphocytes, contribute to the control of viral infection [6]. In terms of antitumour capacity, NK cell-derived IFN-γ can inhibit tumorigenesis and reduce tumour burden by inhibiting angiogenesis [36], increasing fibronectin expression [37], inducing tumour-specific T cell responses [38], and so on. A subset of cancer cells may disseminate from the primary tumour and escape systemic therapy, which would initiate future metastases. One recent study also revealed that the production of IFN-γ by NK cells in local tissue is also critical for inducing quiescence of the disseminated tumour cells, thus preventing their switch from dormancy to outgrowth in the local tissue[39]. Several studies have demonstrated that NM inhibits the proliferation, adhesion, and invasion of several types of cancer cells, such as pancreatic, gastric, and colorectal cancer cells [40–43]. All these findings suggest that NM is a promising candidate for preventing and treating viral infection or cancer.

Our finding that NM could only increase the proportion of IFN-γ-producing NK cells in the NM-treated PBMC population and in the enriched NK cell population but not in the purified NK cell population is interesting. There are some possible explanations for these results. First, in the presence of IL-12 or IL-15, the interaction between NK cells and other lymphocytes, especially T cells and monocytes, that are activated by NM plays a critical role in promoting NK cell IFN-γ production in the PBMC population [44, 45]. In enriched NK cells, there are also some contaminating T cells and monocytes. One previous study has shown that by treating human PBMCs with NM, NM can induce dramatic production of several cytokines, including IL-12, IL-18, IFN-γ, and TNF-α, and induce the expression of CD40, CD40 ligand, B7.1, B7.2, and intercellular adhesion molecule-1 on monocytes [46]. Second, the strong induction of IFN-γ production by IL-12 or IL-15 may mask the synergistic effect of NM and endogenous IL-18. Our data showed that NM alone or in the presence of IL-18 could enhance IFN-γ production by purified NK cells; however, this effect is moderate. In our data, the concentration of IFN-γ in the supernatants of NM-treated and NM plus IL-18-treated purified NK cells was less than 50 and 150 pg/mL, respectively. However, only IL-12 or IL-15 can induce the secretion of up to ~ 500 and ~ 200 pg/mL IFN-γ in the supernatants of purified NK cells after stimulation. This result indicates that NM could not reach the threshold of IFN-γ production when used in combination with IL-12 or IL-15. The detailed mechanisms still warrant further investigation.

Our data showed that NM treatment did not affect the overall cytotoxicity of NK cells, although it reduced the protein levels of CD107a and FASL. Consistently, NM also did not affect the protein levels of NK cell-activating or inhibitory receptors, including NKp30, NKG2D, CD16, and NKG2A. Degranulation and activation of death receptor-related apoptosis via the production of TRAIL and FasL represent different mechanisms by which NK cells exert their cytotoxic effects [24]. TRAIL has become a potential therapeutic target in cancer therapy [47] and plays an important role in the antiviral response, such as the response to dengue infection [48]. The effect of reduced CD107a and FASL expression on NK cell cytotoxicity may be compensated by the enhanced expression of TRAIL. However, we could not exclude the possibility that the NM-mediated increase in TRAIL expression occurred as a result of negative feedback from the downregulation of CD107a expression. One previous study revealed that after ischaemia–reperfusion injury, TRAIL-Null mice have increased numbers of CD107a-positive NK cells compared with wild-type mice [49].

NM selectively activates NK cell IFN-γ production without affecting NK cell cytolytic function. Interestingly, separation of these functions exists naturally in the human immune system, as CD56^bright^ NK cells exhibit relatively higher IFN-γ production, while CD56^dim^ NK cells exhibit relatively higher cytotoxicity [50]. For example, almost all uterine NK cells are CD56^bright^ NK cells, and they are critical for the decidualization and spiral arteriole remodelling that meet the nutritional needs of the foetus [51]. NK cell activation by cytokine stimulation usually enhances both IFN-γ production and cytotoxicity. However, in some contexts, cytotoxicity may cause damage to normal tissues (e.g., in graft-versus-host disease, pregnancy, and liver injury) [52–55]. Interestingly, we found that NM also decreased FASL expression. The expression of both FasL and Fas has been found to be increased on lymphocytes, including NK cells and T cells, from systemic lupus erythematosus patients [56] and is correlated with organ damage and lymphocyte apoptosis [57]. This suggests that NM may also act as a beneficial immune modulator for autoimmune disease treatment that does not obviously affect the capacity of immune surveillance against viral infection or malignant cells. Therefore, its selective induction of NK cell IFN-γ production may provide a way to separately target the two major functions of NK cells, namely, cytokine production, and cytotoxicity.

In conclusion, the current study identifies NM as an effective stimulator of NK cell IFN-γ production. NM is a clinically approved medicine with relatively good safety, and additional studies are warranted to understand the roles and detailed mechanisms by which NM functions in the prevention and treatment of infection or cancer in select populations.

## Supplementary Information

Below is the link to the electronic supplementary material.

Supplemental Fig. 1 Scatter plot of the change in the percentage IFN-γ-producing NK cells in the PBMC population after treatment with 513 metabolism-related compounds. Distribution of the change in the percentage of IFN-γ+ NK cells in the PBMC population after treatment with the 513 active compounds in the presence of IL-12 (10 ng/mL) (A) or IL-15 (10 ng/mL) (B). The change in the percentage of IFN-γ+ NK cells treated with each compound was normalized to DMSO treatment of cells from the same donor.
Supplemental Fig. 2 Effects of the nine identified candidates on IFN-γ secretion by CD3^+^ T cells. PBMCs were treated with DMSO control or one of the nine compounds for 18 h in the presence of IL-12 (10 ng/mL) (A) or IL-15 (10 ng/mL) (B), and the cumulative frequencies of IFN-γ-producing CD3^+^CD56^−^ T cells were analysed by flow cytometry (n = 3). The data were analysed with a paired t test. *, p < 0.05; **, p < 0.01; ns (not significant), p >  0.05 compared with the DMSO control group.
Supplemental Fig. 3 Effects of NM on IFN-γ-producing NK cells in enriched NK cells. (A) Enriched NK cells were treated with DMSO (control) or NM (0, 5, or 25 μM) for 24 h in the presence of IL-12 (10 ng/mL) or IL-15 (10 ng/mL), and the proportion of IFN-γ-producing NK cells was assessed by flow cytometry (n=9). (B) Enriched NK cells were treated with DMSO or NM for 6 h in the presence of IL-12 (10 ng/mL) or IL-15 (10 ng/mL), and the protein levels of IFN-γ in the cell culture supernatants were measured with an ELISA kit (n = 3). The data were analysed by the Friedman test and are shown as the median ± interquartile range. Each of the dot colours represents matched data from the same donor. *, p < 0.05; **, p < 0.01; ns (not significant), p > 0.05 compared with the DMSO control group. (C) The percentage of IFN-γ+ cells in both the CD56bright and CD56dim NK cell populations in PBMCs treated with NM in the presence of IL-12 (10 ng/mL). The data were analysed with a paired t test. *, p < 0.05.
Supplemental Fig. 4 Effects of NM on IFN-γ secretion by T cells and monocytes in enriched NK cell samples. (A) Flow cytometry was used to analyse the proportions of CD3^+^ and CD14^+^ cells in enriched NK cell samples. (B) Enriched NK cells were treated with DMSO or NM for 24 h in the presence of IL-12 (10 ng/mL) or IL-15 (10 ng/mL), and the proportions of IFN-γ-producing T cells and monocytes were determined by flow cytometry.
Supplemental Fig. 5 Effects of nafamostat mesylate on cytotoxicity and expression of germline-encoded receptors. Representative flow plots and cumulative frequencies of cells expressing CD107a (A), TRAIL, and FASL (B) and NKp30, NKG2D, CD16, and NKG2A (C) for the corresponding data in Figure 4A, B and D are shown. The data were analysed with a paired t test.
Supplementary file1 (PDF 1401 kb)

## Data Availability

The authors declare that all the original materials for this study will be provided upon request.
