# Supplementary figures and images for: Identification of nafamostat mesylate as a selective stimulator of NK cell IFN-γ production via metabolism-related compound library screening
Source: Immunol Res. 2022 Feb 15;70(3):354–64. doi: 10.1007/s12026-022-09266-z (PMC8852993; doi:10.1007/s12026-022-09266-z)

Fig S1

A

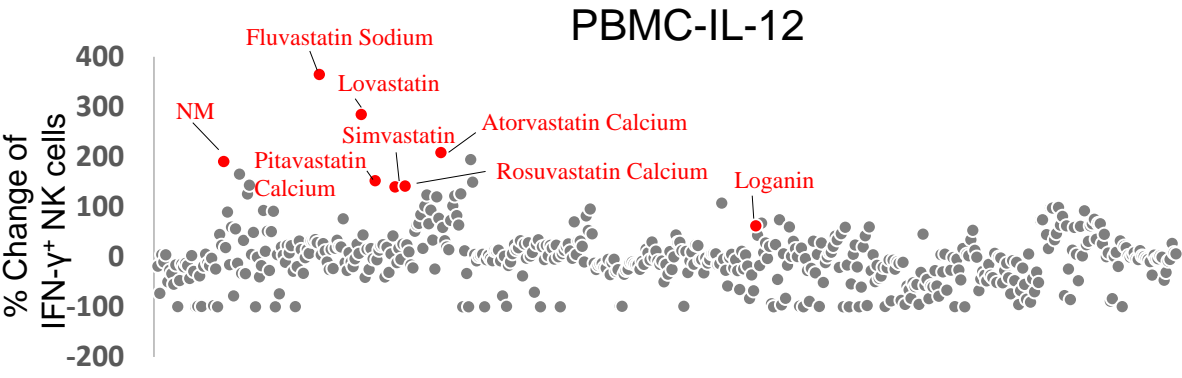

B

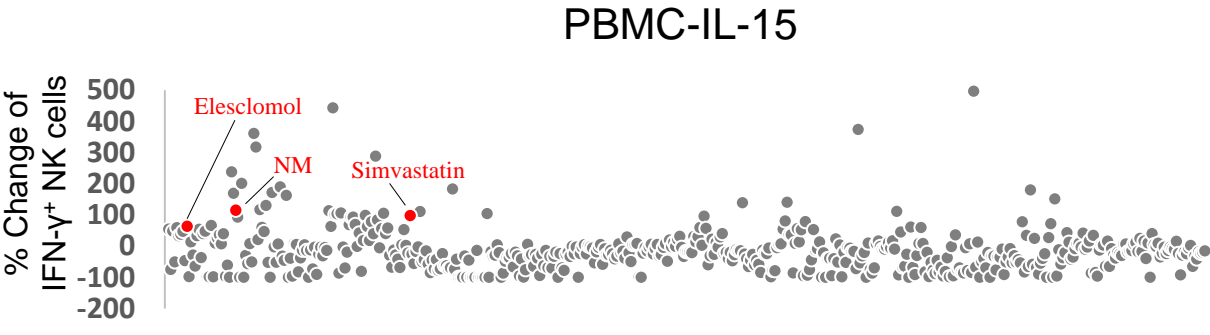

Fig S2

A

PBMC-IL-12

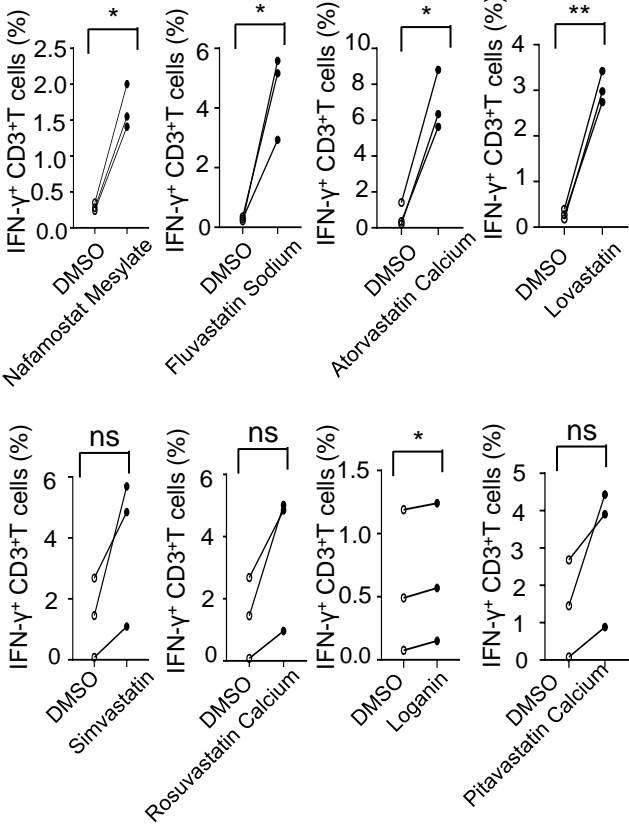

B

PBMC-IL-15

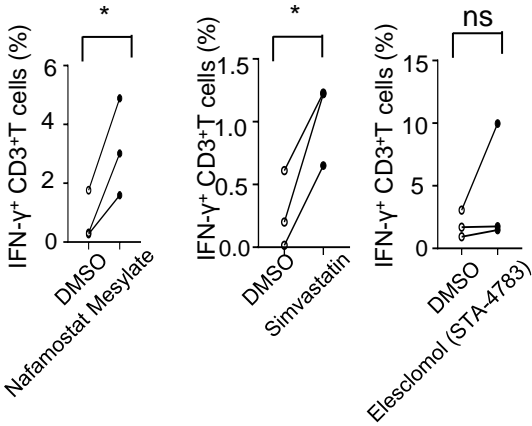

Fig S3

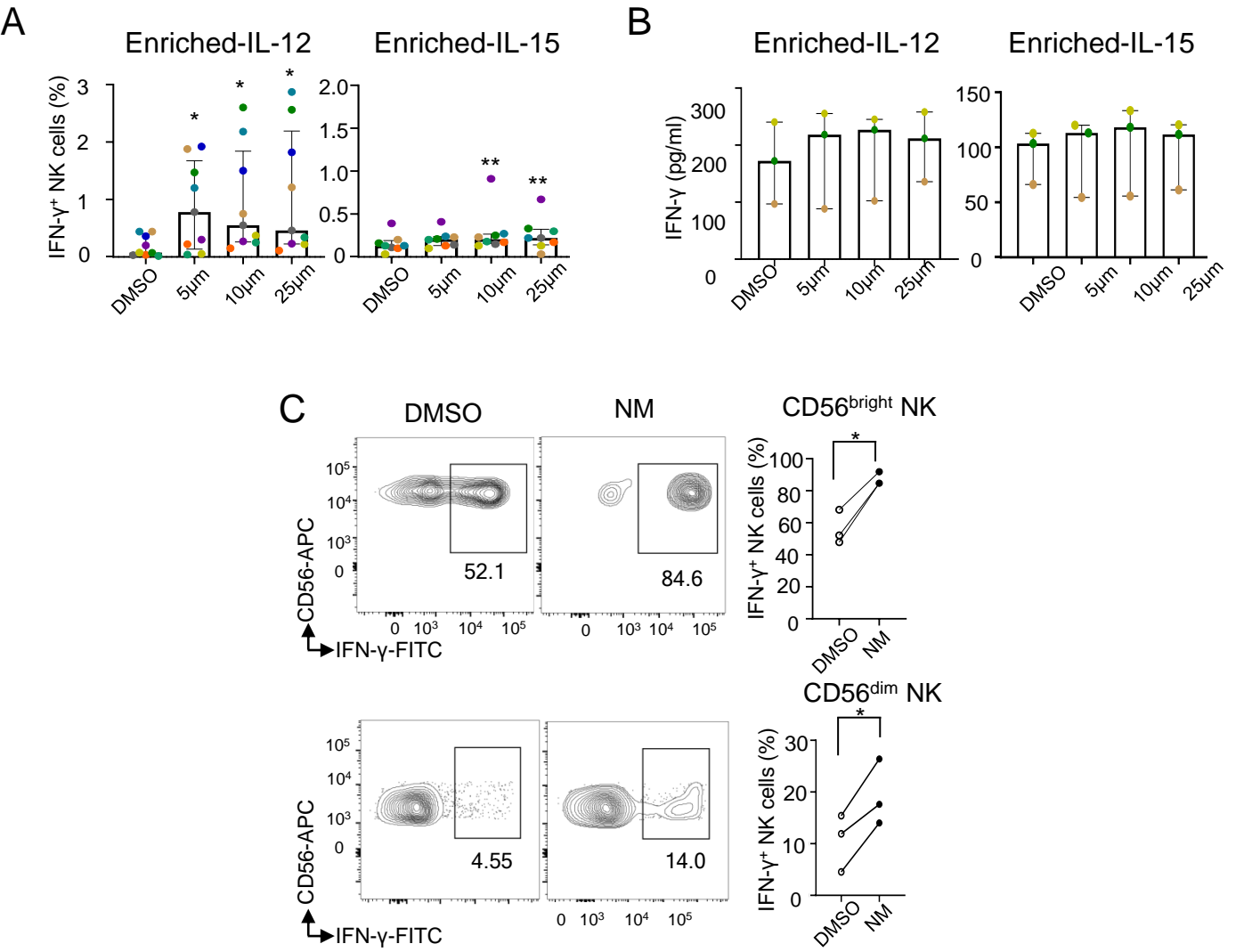

Fig S4

A

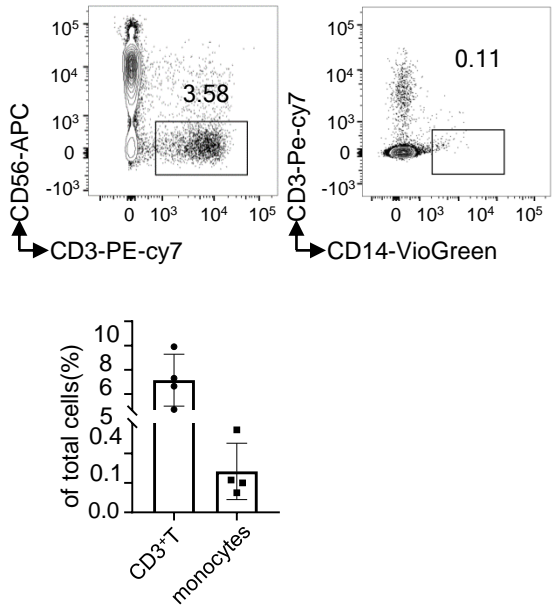

B

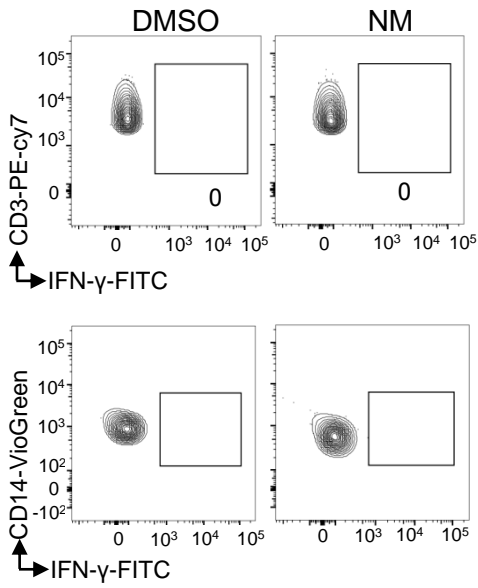

Fig S5

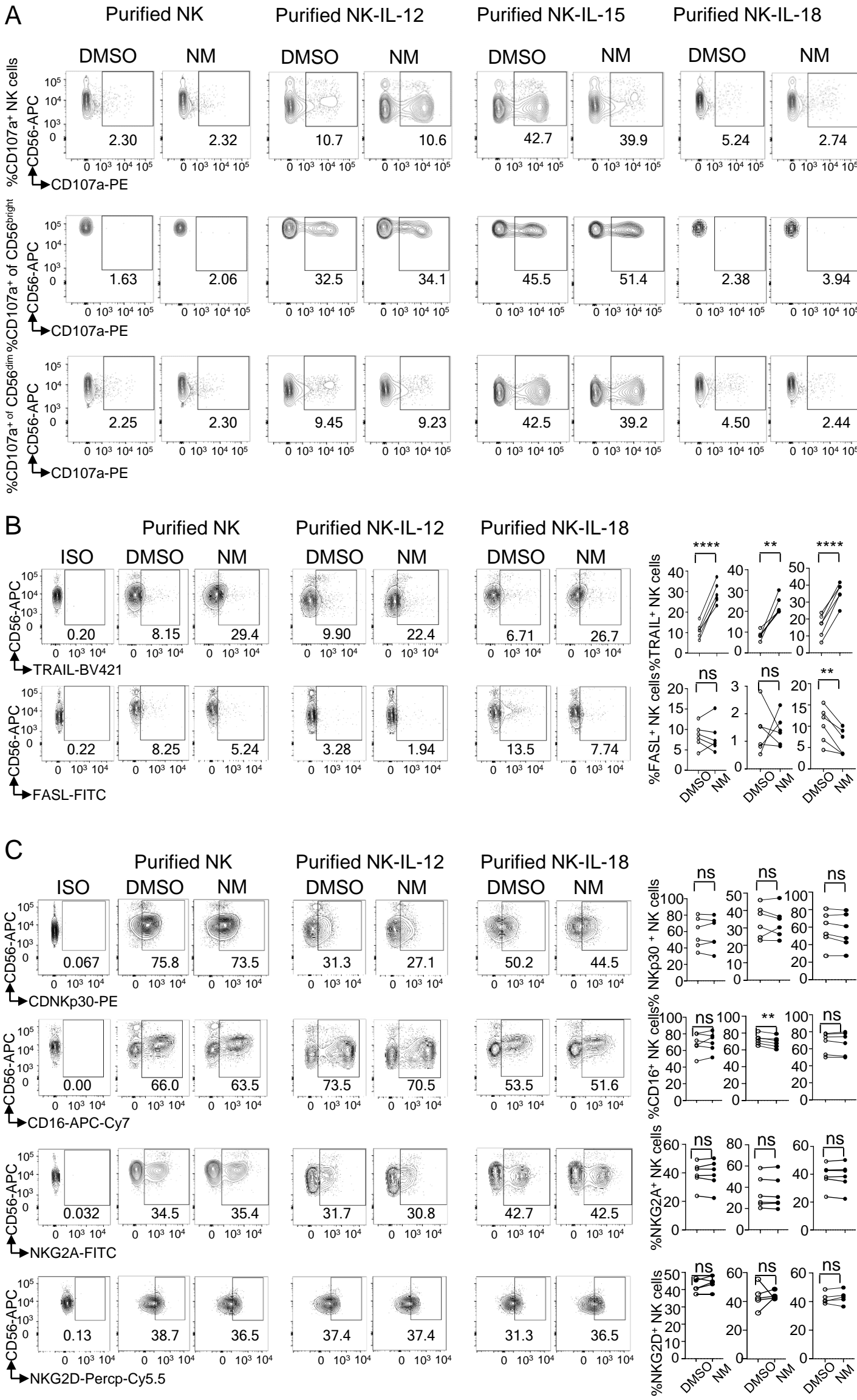

Supplement: Supplementary file 1 — Supplemental Fig. 1 Scatter plot of the change in the percentage IFN-γ-producing NK cells in the PBMC population after treatment with 513 metabolism-related compounds. Distribution of the change in the percentage of IFN-γ+ NK cells in the PBMC population after treatment with the 513 active compounds in the presence of IL-12 (10 ng/mL) (A) or IL-15 (10 ng/mL) (B). The change in the percentage of IFN-γ+ NK cells treated with each compound was normalized to DMSO treatment of cells from the same donor. Supplemental Fig. 2 Effects of the nine identified candidates on IFN-γ secretion by CD3+ T cells. PBMCs were treated with DMSO control or one of the nine compounds for 18 h in the presence of IL-12 (10 ng/mL) (A) or IL-15 (10 ng/mL) (B), and the cumulative frequencies of IFN-γ-producing CD3+CD56− T cells were analysed by flow cytometry (n = 3). The data were analysed with a paired t test. *, p < 0.05; **, p < 0.01; ns (not significant), p > 0.05 compared with the DMSO control group. Supplemental Fig. 3 Effects of NM on IFN-γ-producing NK cells in enriched NK cells. (A) Enriched NK cells were treated with DMSO (control) or NM (0, 5, or 25 μM) for 24 h in the presence of IL-12 (10 ng/mL) or IL-15 (10 ng/mL), and the proportion of IFN-γ-producing NK cells was assessed by flow cytometry (n=9). (B) Enriched NK cells were treated with DMSO or NM for 6 h in the presence of IL-12 (10 ng/mL) or IL-15 (10 ng/mL), and the protein levels of IFN-γ in the cell culture supernatants were measured with an ELISA kit (n = 3). The data were analysed by the Friedman test and are shown as the median ± interquartile range. Each of the dot colours represents matched data from the same donor. *, p < 0.05; **, p < 0.01; ns (not significant), p > 0.05 compared with the DMSO control group. (C) The percentage of IFN-γ+ cells in both the CD56bright and CD56dim NK cell populations in PBMCs treated with NM in the presence of IL-12 (10 ng/mL). The data were analysed with a paired t test. *, p < 0.05. Sup [file 12026_2022_9266_MOESM1_ESM.pdf]
